# A complex network approach to political analysis: Application to the Brazilian Chamber of Deputies

**DOI:** 10.1371/journal.pone.0229928

**Published:** 2020-03-19

**Authors:** Ana Caroline Medeiros Brito, Filipi Nascimento Silva, Diego Raphael Amancio

**Affiliations:** 1 Institute of Mathematics and Computer Science, University of São Paulo, São Carlos, SP, Brazil; 2 São Carlos Institute of Physics, University of São Paulo, São Carlos, SP, Brazil; 3 Indiana University Network Science Institute, Bloomington, Indiana, United States of America; Consejo Nacional de Investigaciones Cientificas y Tecnicas, ARGENTINA

## Abstract

In this paper, we introduce a network-based methodology to study how political entities evolve over time. We constructed networks of voting data from the Brazilian Chamber of Deputies, where deputies are nodes and edges are represented by voting similarity among deputies. The Brazilian Chamber of deputies is characterized by a multi-party political system. Thus, we would expect a broad spectrum of ideas to be represented. Our results, however, revealed that plurality of ideas is not present at all: the effective number of communities representing ideas based on agreement/disagreement in propositions is about 3 over the entire studied time span. The obtained results also revealed different patterns of coalitions between distinct parties. Finally, we also found signs of early party isolation before presidential impeachment proceedings effectively started. We believe that the proposed framework could be used to complement the study of political dynamics and even applied in similar social networks where individuals are organized in a complex manner.

## Introduction

In recent years, the availability of information in the form of open datasets together with the capabilities to store and process data have been promoting the rise of many new studies in a variety of disciplines, including Biology [1], Social Sciences [2], Linguistics [3–6] and Physics [7, 8]. Many of these systems can be regarded as being too complex for traditional methodologies in which each of its components is isolated and studied individually. This includes the political dynamics of a country, which may depends on many aspects such as economic factors, culture, mass media, social media, etc. In a democratic system, these aspects are reflected (or should be) on the decisions made by people’s representatives and on how they are organized (such as partisanship or alliances between parties). In this context, the relationships between politicians could be drawn indirectly from how they vote in propositions [9–11], i.e., their agreement or disagreement among a set of voted proposals.

Politicians with similar voting patterns can be understood as having similar views and interests, thus can be connected in a political network. Being a complex system, it makes sense to explore such a system by using methods borrowed from network science [12]. The Brazilian political environment becomes an interesting system to be studied under this approach since it is a multi-party system, thus, supposedly presenting a diverse spectrum of political ideas. Also, it is a system that has undergone many changes over the past few years. Moreover, data for decisions and political organization are openly available.

In this paper, we propose a framework based on complex networks to study the evolution of the Brazilian political system in terms of how politicians vote in proposals along time. For that, we collected and processed voting data from 1991 to 2018, spanning 6 terms in the lower chamber and build vote correlation networks. Because the obtained networks were weighted and dense, we applied a filtering method to preserve the community structure. Using concepts borrowed from network science, we defined relevant political concepts, including *coalition*, *fragmentation* and *isolation*. We also devised a measure to quantify the variability in the size of groups obtained in the generated political networks.

Several interesting results were obtained in the Brazilian political scenario. We found in most years a low correspondence between communities (i.e. topological groups) and political parties, meaning that the majority of deputies tend to share votes with different parties. Most importantly, despite the large number of political parties in the Brazilian political scenario (29 parties in 2019), only roughly 3 network communities were identified. The quantification and visualization of shared coalitions and isolation were consistent with the political scenario unfolded along the considered period. We also found different behavior of coalitions: while some parties tend to be aligned to the president’s party, other parties preserve their voting behavior, regardless of who is the current president. Finally, we identified an isolation pattern of president’s party in a short period before the impeachment process effectively started. Such an isolation was characterized by a group of deputies displaying a voting behavior different from all other parties. This segregation behavior could be an early indication of the start of an impeachment process. While we focused on the analysis of the Brazilian case, we advocate that the methods are robust enough to be applied in other democratic systems and even in other social networks.

## Related works

Some studies used the concept of complex networks to model some political concepts, including the concept of political *coalition* and *cohesion* [10, 11, 13, 14]. Both concepts are quantified via networks extracted from parliamentary votes.

In [10], network edges are established according to the similarity of votes between parliamentarians. Votes were considered to be chosen among three options. Deputies can vote for and against a specific proposition. In addition, they may abstain from voting. All data were extracted from the Italian parliament in 2013. The authors define cohesion in terms of the link density among the deputies in the same party compared to those in different parties. The cohesion defined in terms of network topology revealed that some parties are well defined, while some parliamentarians display a voting pattern that is most similar to the patterns observed in other parties.

Also, network communities and relevant parliamentarians were identified by measuring the effect of removing and inserting nodes in distinct network communities. More specifically, the authors measured the variation in the modularity d*Q* [15] when parliamentarians with distinct political positions are placed in their respective opposite political communities. The degree of polarization was then defined as proportional to the absolute variation |d*Q*| resulting from this procedure.

Upon analyzing the modularity of the networks, the authors advocate that it may not be the best alternative to characterize the obtained clustered networks. Their analysis also showed the emergence of three main communities, where two communities represent parliamentarians voting according to the government preferences, while the remaining community was found to represent opposition parties. Finally, a temporal analysis revealed patterns of voting behavior along time. Such a temporal analysis was useful to identify e.g. parties do not display a consistent voting behavior.

In [13], a dataset of propositions analyzed between 2011 and 2016 was used to analyze votes in the Brazilian political scenario. Nodes were defined as deputies, which are linked by the number of identical votes in the considered period. Different variations in the network construction were considered because abstentions may lead to different interpretations on whether two deputies agree when at least one of them abstain from voting. This study also defined political concepts such as polarization in social networks using correlation clustering [16] and its symmetric relaxed version to quantify polarization.

The analysis carried out in [13] revealed that several parties do not comply with their respective original ideas as expressed during the campaign period. In addition, this study showed that, in recent years, the group of deputies allied to the Brazilian government diminished. Even after presidential reelection, allied deputies turned out to display a voting pattern in disagreement with government propositions. Finally, this study reports that the distance between centre- and right-wing deputies decreased in recent years.

The research conducted in [14] studied the network community organization of parliamentarians, focusing on their temporal evolution. The data were obtained from both Brazilian Chamber of Deputies and US Parliament from 2003 to 2017. Temporal networks were created without overlapping. In the network representation, parliamentarians are nodes and edge weights are established according to the vote similarity. In the Brazilian case, considered two additional possibilities for each vote: (i) abstention; and (ii) obstruction. In the latter, it may also be considered as a vote against the proposition under discussion. The authors proposed a measurement that depends on the community structure of the network. The *Partisan Discipline*, computed for a given parliamentarian *m*, was defined as

$$\text{pd}\left(m\right)=\frac{1}{N}\sum_{i=1}^{N}I\left(m,p_{m},i\right),$$ (1)

where *N* is the total number of propositions analyzed and *I*(*m*, *p*_*m*_, *i*) = 1 iff *m* and the respective group (i.e. network community or party) to which he/she belongs voted equally in the *i*-th proposition.

The application of Eq 1 for the data obtained from the Brazilian Chamber of Deputies revealed that the values of *Partisan Discipline* are higher when deputies are clustered in network communities in comparison to clusters being political parties. For the US Parliament, the obtained values of *Partisan Discipline* are similar when parties and communities are considered. The concept of polarization was analyzed by observing that cohesive communities emerges in a political scenario characterized by a high degree of polarization. Because members of the same community should have similar sets of neighbors, the number of shared neighbors was used as an indicative of political polarization. The polarization analysis revealed that the Brazilian Chamber of Deputies has a low degree of polarization. In addition, this study showed that deputies oftentimes do not stay in the same dense, well-defined community for long periods.

Some studies used political networks to identify the ideology of political parties. While many studies focused on voting data, the study reported in [17] focused on party-switching affiliations from one election to another to generate networks. In such a network, nodes are parties and two parties are linked if there was a migration of deputies between these parties. The community structure of the obtained networks revealed a tendency of deputies to switch between parties with similar ideology. A model was also created to quantify the ideology of political parties on the left-right scale. The methodology was found to be robust as it does not depend on the level of representation of parties, since only affiliation data is required to create political networks.

The European Parliament was studied using networks in the work conducted in [11]. A dataset with roll-call voting data from the European Parliament was used to create a network where parliamentarians are nodes and edges are weights according to the similarity of voting patterns. Twitter was also used to create a network where parliamentarians are connected if there is a “retweet” relationship between them. The main results revealed the emergence of political coalitions, characterized by cooperation of opposite political wing groups. In addition, they found a tendency of cooperation among small and large political groups. Finally, this study also reports that political alignments in Twitter are consistent with voting patterns.

Complex networks have also used to analyze political process in different contexts. In [18], socio-political processes behind the 2016 US election were studied. A network of right-ring users was created so that two users are linked whenever both follow the same user. A community detection strategy was used to detect groups of similar users and the most relevant words were extracted using a strategy similar to tf-idf [19]. The authors found an increase in the number of users supporting Donald Trump and a change of behavior of republican users. Network science was also used to model the competition of candidates to gain votes in a social network [20]. Using a model of a random network model as a example of social network, Melo *et al*. studied how economical capacity can be used to influence voters. The authors found that the candidates receiving the highest number of votes usually spend more economical resources per vote.

Unlike the above mentioned studies, we propose metrics based on shortest path lengths which can be understood as the connection strength between parliament members. We also use temporal series to show our results, enabling thus the visualization of political parties dynamics. Differently from other works, we compare the structure of political parties with the structure observed from voting behavior. Our focus is on the characterization of parties, which encompasses the definition of political fragmentation, isolation and diversity of parties.

## Methodology

The proposed *framework* to analyze the relationship among deputies according to their voting patterns can be summarized in the following steps:

1. *Network construction*: a network is created from voting patterns in a given period. Two deputies are linked if they vote in a similar fashion in several propositions.
2. *Network backbone extraction*: this step involves the removal of the weakest edges. This is essential to create a network that can be treated by most of the traditional community detection methods. Mostly important, the removal of the weakest edges does not affect the community structure of the obtained networks.
3. *Community detection and analysis*: community detection methods are employed to identify groups of deputies displaying similar voting patterns.
4. *Network characterization*: metrics are extracted from the network in order to quantify political concepts such as fragmentation and isolation. We also use a measure to quantify the variability in the size of political parties and network communities.

**Fig 1:**
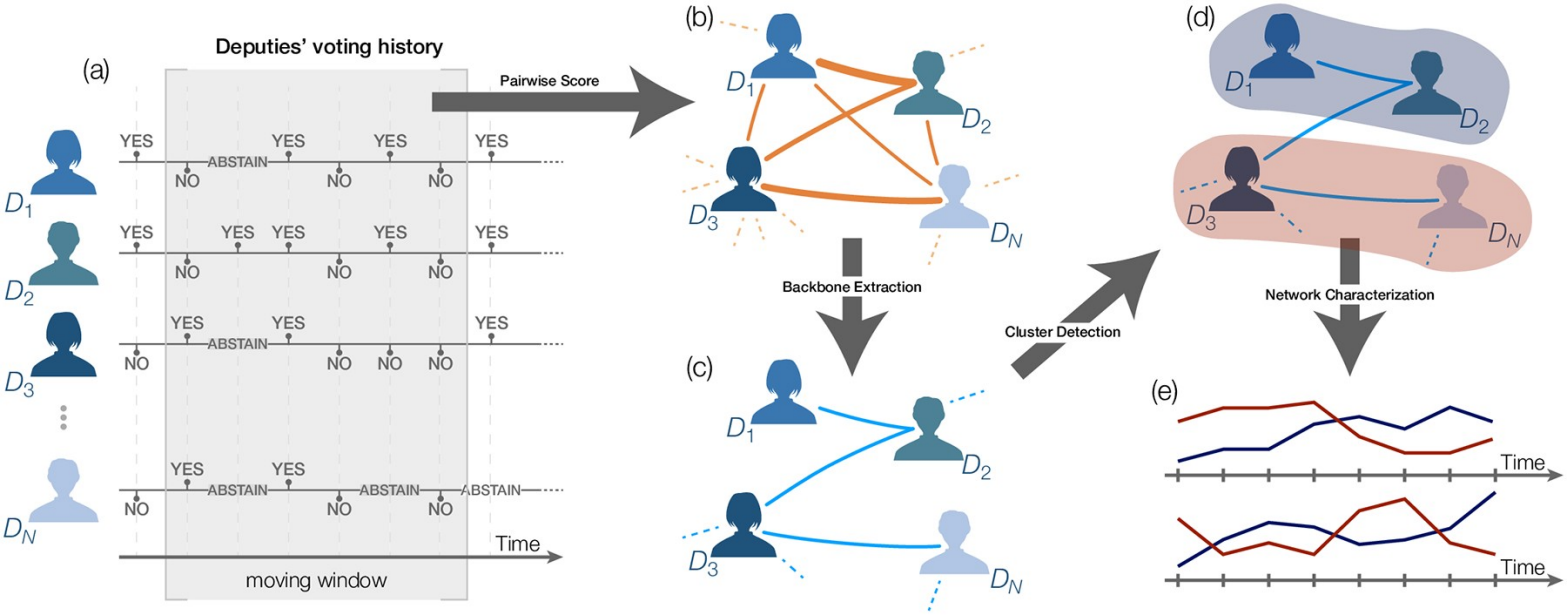
Schematic representing the methodology in this paper. In (a), the voting data is analyzed. In (b), the pairwise similarity of votes in distinct propositions generates a weighted network of deputies. Edges weight represent the distance between two deputies, according to their voting behavior. In (c), edges are filtered so as to preserve the community structure. In (d), we identify the community structure of the network. Finally, in (e), the resulting network is analyzed by measuring e.g. distance between parties.

These four steps are detailed, respectively, in the next subsections. All involved steps are illustrated in Fig 1.

### Network construction

Networks are constructed by representing deputies as nodes. Edges are established between two deputies if the percentage of agreement considering all propositions in the dataset is higher than the percentage of disagreement. Mathematically, all votes established by the *i*-th deputy can be stored in a *N*-dimensional vector **v**^(*i*)^, where *N* is the total number of considered propositions. The *j*-th element of **v** may store three different values. **v**_*j*_ may take the values −1 or + 1, representing respectively a vote for or against a given proposition. In case of abstention, **v**_*j*_ = 0. Thus, the pairwise level of agreement between two deputies is defined as:

$$w_{ij}=\frac{1}{N}\sum_{k=1}^{N}v_{k}^{\left(i\right)}v_{k}^{\left(j\right)}=\frac{v^{\left(i\right)}\cdot v^{\left(j\right)}}{N},$$ (2)

where **v**^(*i*)^ ⋅ **v**^(*j*)^ is the dot product between **v**^(*i*)^ and **v**^(*j*)^. Note that, when deputies have the same opinion about the proposition being voted, the level of agreement is increased by 1. Conversely, when views are opposite, *w*_*ij*_ is decreased by 1. Whenever at least one of the deputies absence from voting in a proposition, that proposition is not considered when computing *w*_*ij*_.

Because edges weights are given by *w*_*ij*_, all obtained networks are undirected and weighted. As we shall show, the networks may be constructed using a particular time interval, and also considering sliding (overlapping) windows of fixed length.

### Network backbone extraction

Even considering only agreement between deputies as connections, the proposed approach can still lead to densely connected networks. While such networks can still be regarded as a weighted, it is usually desirable to remove noise from data, such as weak connections that may correspond to spurious links. For that reason, we included a preprocessing step to our analysis pipeline in which we deal with the problem of keeping only important connections in a network. Such a task is known as edge pruning [21].

A simple way to accomplish edge pruning is keeping only edges with weights larger than a fixed threshold. Such a threshold can be determined, for instance, in terms of percentiles to reach a certain network density. The main problem with that approach is that it may introduce some bias toward highly connected nodes which can lead to substantial undesired effects on the network topology.

A more sophisticated approach to the edge pruning task is the backbone extraction method [22]. This technique is based on the idea that when determining the importance of an edge, we must take into account how its ending points are connected to the network. More specifically, the authors proposed that the importance of a node can be determined in terms of a disparity filter, so that a value *α*_*ij*_ for an edge can be drawn from a *p*-value determined in terms of null model. This null model considers the probability of a node having an edge with a certain weight based on its other connections. In practice, one can compute *α*_*ij*_ for a certain edge *e*_*ij*_ existing in the network as:

$$\alpha_{ij}=1-\left(k_{i}-1\right)\int_{0}^{p_{ij}}{\left(1-x\right)}^{k_{i}-2}dx,$$ (3)

where *k*_*i*_ is the number of connections for node *i* and *p*_*ij*_ is a probability defined as

$$p_{ij}=\frac{w_{ij}}{\sum_{ik\;\in \;E}w_{ik}}.$$ (4)

The resulting network is obtained by removing all edges with *α*_*ij*_ higher than a certain threshold *α*. Because the networks under analysis are undirected, we consider, for each edge *e*_*ij*_, the lowest between *α*_*ij*_ and *α*_*ji*_. Here, we opted no to use a fixed threshold for *α*. We instead used the smallest *α* such that each network have at least 80% of the nodes belonging to the giant component. This results in more consistent networks along time and allow us to analyze the data based on the major component. This is important in network analysis because some measurements, such as shortest path distances, are only measured in a connected component.

**Fig 2:**
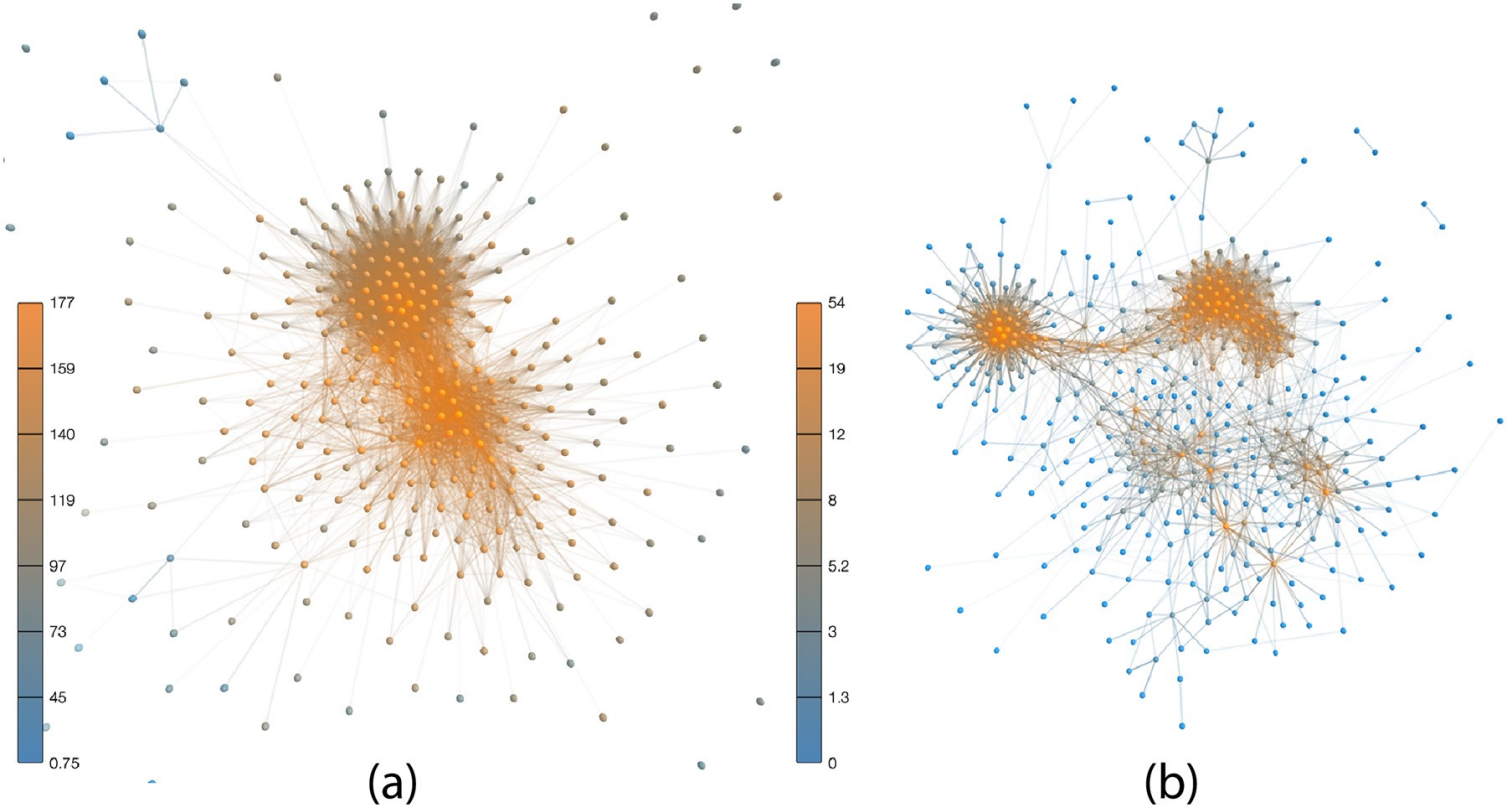
Network obtained considered all proposition voted in 2012. The networks were constructed (a) *without* edge pruning; and (b) *with* edge pruning. Each node represents a member from the Brazilian lower chamber. Two members are linked if they voted in a similar way. Colors represent nodes degree. This visualization was created using the *Networks3D* visualizer software [19].

In order to illustrate the edge pruning process, we show in Fig 2 a visualization of a network before and after the pruning process is applied. Interestingly, there is a clear separation in communities of deputies with very distinct voting patterns. The emergence of several communities is not as clear when the filtering procedure is not applied.

### Community detection and analysis

Once the network is obtained, nodes are grouped into communities to allow us to understand how deputies are clustered according to voting behavior. The identification of communities is also important to analyze if the organization of deputies in parties is consistent with the natural organization emerging from their voting behavior.

Several methods rely on the *modularity* to measure the quality of the obtained communities in complex networks [23, 24]. This measurement quantifies the number of links that occur inside a community that are above the same number expected in a null model. Mathematically, the network modularity *Q* is given by

$$Q=\frac{1}{2m}\sum_{ij}[a_{ij}-\frac{k_{i}k_{j}}{2m}]\delta \left(c_{i},c_{j}\right),$$ (5)

where *a*_*ij*_ = 1 if nodes *i* and *j* are connected and *a*_*ij*_ = 0, otherwise. *m* = 1/2∑_*ij*_ *a*_*ij*_ is the total number of edges in the network and *k*_*i*_ = ∑_*j*_ *a*_*ij*_. *δ*(*c*_*i*_, *c*_*j*_) = 1 only if nodes *i* and *j* are in the same community. Otherwise, *δ*(*c*_*i*_, *c*_*j*_) = 0.

In this particular study, we used the Leiden method [25], which is an improved version of the Louvain algorithm [23]. While we report our results based on this Leiden method, a preliminary study revealed that the choice of community detection method has no influence on the results reported in this paper.

The Louvain algorithm is based on the iterative repetition of two steps. First, each single node is considered as a single community in the network. Then, for each node, the method evaluates the gain in modularity when it is removed from its current community and placed to a neighbor community. The change of community membership is effectively performed if such a change corresponds to the highest gain in modularity among all possible changes in the current iteration. If the highest gain is negative (i.e. the change of community membership leads to a decrease in modularity), then the node stays in its original community. This process is repeated until no gain in modularity is achieved. The efficiency of the method stems from the fact that the gain of modularity can be computed in a efficient way. The algorithm proposed in [25] guarantees that the obtained communities are connected.

One particular feature that we are interested in the study of political networks is the correspondence between political parties and network communities. We are particularly interested in measuring the effective number of groups, where groups are either political parties or topological communities. A more refined notion of group diversity (i.e. total number of groups) in a partition may be derived from the concept of *true diversity* [26]. This quantity measures the effective number of groups in a partition by considering the size of groups. Let $S=\{s_{1},s_{2},\ldots s_{R}\}$ be the size of each group in a partition comprising *R* groups. The computation of the true diversity requires that the sizes in S are normalized. For this reason, they are normalized to yield the following set of normalized sizes Π = {*π*_1_, *π*_2_, …*π*_*R*_}, where *π*_*i*_ = *s*_*i*_/∑_*j*_ *s*_*j*_. The true diversity *D*^(*q*)^ of the distribution Π is defined as

$$D^{\left(q\right)}=(\sum_{i=1}^{R}\pi_{i}^{q}{)}^{1/(1-q)},$$ (6)

where *q* is the exponent given to the weighted generalized mean of *π*_*i*_’s. If *q* = 1, the true diversity can be computed as

$$D^{\left(q=1\right)}=\underset{q\to 1}{\text{lim}}(\sum_{i=1}^{R}\pi_{i}^{q}{)}^{1/(1-q)}=\frac{1}{\prod_{i=1}^{R}\pi_{i}^{\pi_{i}}}=\text{exp}(-\sum_{i=1}^{R}\pi_{i}\text{ln}\pi_{i}),$$ (7)

which corresponds to the exponential of the entropy of Π. The true diversity has been employed in other contexts to measure the *effective* number of species [27]. In network theory, the accessibility of nodes in complex networks is measured in terms of the true diversity and quantify the *effective* number of neighbors [28–33]. Because we are measuring the diversity of communities (or parties) size, hereafter we refer to the true diversity as the *effective* number of political parties and communities.

### Network characterization

In this section, we introduce the measurements that are used to analyze network obtained from voting patterns. We are specially interested in the analysis of distances among parties along time. The first important measurement that can be extracted from political networks is the topological distance *d*(*A*, *B*) between two parties *A* and *B*, defined as the average distance among their elements:

$$d\left(A,B\right)=\frac{1}{\left|A\times B\right|}\sum_{(a,b)\in A\times B}l\left(a,b\right).$$ (8)

where *l*(*a*, *b*) is the shortest path length between *a* ∈ *A* and *b* ∈ *B*. The distance *d*(*A*, *B*) can be used to quantify the level of *coalition* between *A* and *B*. In this case, low values of distances correspond to strong coalitions.

Another important quantity is the average distance *d*_*G*_ between all nodes in the network. It is defined as:

$$d_{G}=\frac{2}{n(n-1)}\sum_{a\neq b}l\left(a,b\right).$$ (9)

Note that the distance *l* can not be computed directly from the network obtained after edge filtering, because edge weights represent a similarity relationship. In order to map the similarity *w*_*ij*_ defined in Eq 2 into a dissimilarity index, we used the following equation:

$$\Delta \left(w_{ij}\right)=[2\cdot \left(1-w_{ij}\right){]}^{1/2}.$$ (10)

A more detailed description on network transformations, such as the one in Eq 10, can be found in [34].

The political *isolation* of group *A*, *I*(*A*), is defined as the distance between group *A* and all other groups in a given division of the network. It is defined as

$$I\left(A\right)=\sum_{X\neq A}\left|X\right|\cdot d\left(A,X\right)\;\;/\sum_{X\neq A}\left|X\right|,$$ (11)

which represents the average distance (as defined in Eq 8) between *A* and all other groups. Note that the distances are weighted by the size of each group *X* ≠ *A*. According to Eq 11, the isolation reflects how distant nodes belonging to the group of reference are from the other nodes in the network. Interestingly, there is an analogy between the isolation of groups and the ability (complexity) of unsupervised classification [35]. In other words, isolated groups are analogous to outlier clusters in unsupervised data analysis.

The concept of political *fragmentation* for group *A*, *F*(*A*), can be defined in terms of intra-group dispersion, i.e.

F(A)=d(A,A). (12)

Therefore, a group is highly fragmented whenever the nodes in that group are distant from each other, according to the topology of the network. Note that the quantities defined in Eqs 11 and 12 are regarded as being independent. Interestingly, the fragmentation concept has also been used in network analysis [36], however with different interpretation in the context of network robustness to attacks.

## Dataset

The voting data were obtained from the Brazilian Chamber of Deputies. The data is available at https://dadosabertos.camara.leg.br. The dataset comprises a total of 3, 530 propositions voted between April, 1991 and Juny, 2019. The most frequent political parties appearing in this dataset are: Brazilian Democratic Movement (*Movimento Democrático Brasileiro*—MDB), Brazilian Social Democracy Party (*Partido da Social Democracia Brasileira*—PSDB), Workers’ Party (*Partido dos Trabalhadores*—PT), Progressive Party (*Partido Progressista*—PP), Democrats (*Democratas*—DEM), Communist Party of Brazil (*Partido Comunista do Brasil*—PCdoB), Brazilian Socialist Party (*Partido Socialista Brasileiro*—PSB), Brazilian Labour Party (*Partido Trabalhista Brasileiro*—PTB), Liberal Party (*Partido Liberal*—PL), Democratic Labour Party (*Partido Democrático Trabalhista*—PDT) and Social Liberal Party (*Partido Social Liberal*—PSL). It is important to note that a few of these parties have undergone splitting or merging processes leading to changes in their names over the past few years. However, for the sake of simplicity, we only consider their most recent names, as of 2019.

**Fig 3:**
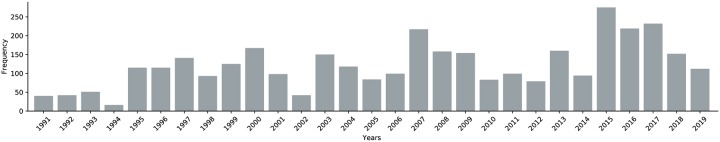
Yearly number of propositions analyzed by the Brazilian Chamber of Deputies between 1991 and 2019. While the number of propositions analyzed along time is not regular, more recently at least 100 propositions were analyzed in each year.

**Fig 4:**
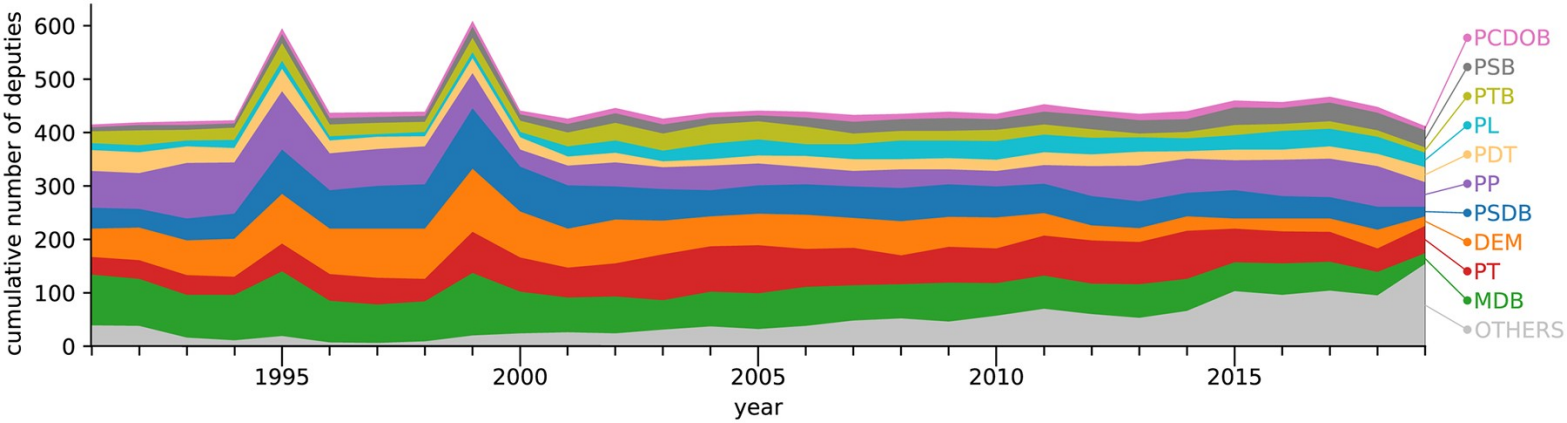
Number of seats of political parties as observed in the Brazilian Chamber of Deputies between 1991 and 2019. The number of deputies from nontraditional parties (“others”) clearly increasing along time. Only the deputies in the largest component of the network were considered.

The total number of propositions voted in each year is shown in Fig 3. In Fig 4, we show the distribution of parties (i.e. the total number of parliamentarians) along time. Interestingly, the representation of nontraditional parties have been increasing along time, while the size of traditional political parties (e.g. MDB and PSDB) is decreasing. A visualization of the obtained network over time is shown in Fig 2. The networks were constructed from voting data for periods from the beginning to the end of each year. We did not consider time windows between different years (even those corresponding to the same term) because the Chamber of Deputies recesses between December 22nd and February 2nd of the following year.

## Results and discussion

Our main objective here is to leverage network science to better understand the dynamics of groups of deputies from a networked perspective. Using the proposed methodology, we intend to address the following general research questions, which are analyzed in the specific case of the Brazilian Chamber of Deputies.

1. Is the organization in parties consistent with network communities formed via voting behavior?
2. Does the diversity of political parties correspond to the diversity of network communities?
3. Is it possible to observe and/or quantify political concepts such as fragmentation and approximation of political parties along time?
4. Is there any signal from network topology indicating the beginning of major political transitions, such as presidential impeachment?

The response to the above questions is important to understand the dynamics of deputies relationships, both at the microscopic and mesoscopic level. Such an better understanding may point out to better ways to improve the democratic system of a country. In the same line, the extraction of information from unstructured date might assist the population to take decisions on who they choose as representatives based on the voting history of deputies and parties alike.

In order to address the above research questions, we divide our analysis in three parts. First, we analyze the global properties of the networks and compare the groups defined via communities and political parties. We also analyze the obtained networks at the mesoscopic (party) level. Lastly, we study how both analyses relate to each other by understanding the distribution of parties among communities.

### Global analysis

**Fig 5:**
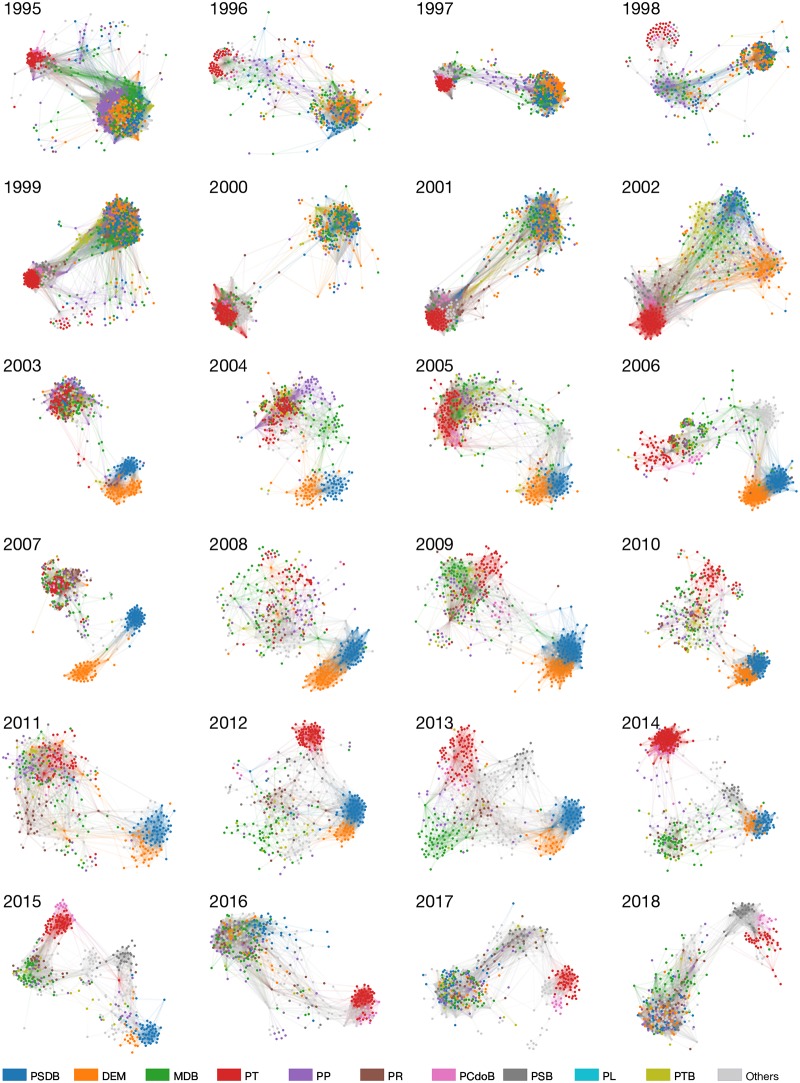
Visualization of the time evolving network obtained from voting data. Each yearly snapshot was obtained considering only the proposals voted in that year. Colors correspond to parties.

**Fig 6:**
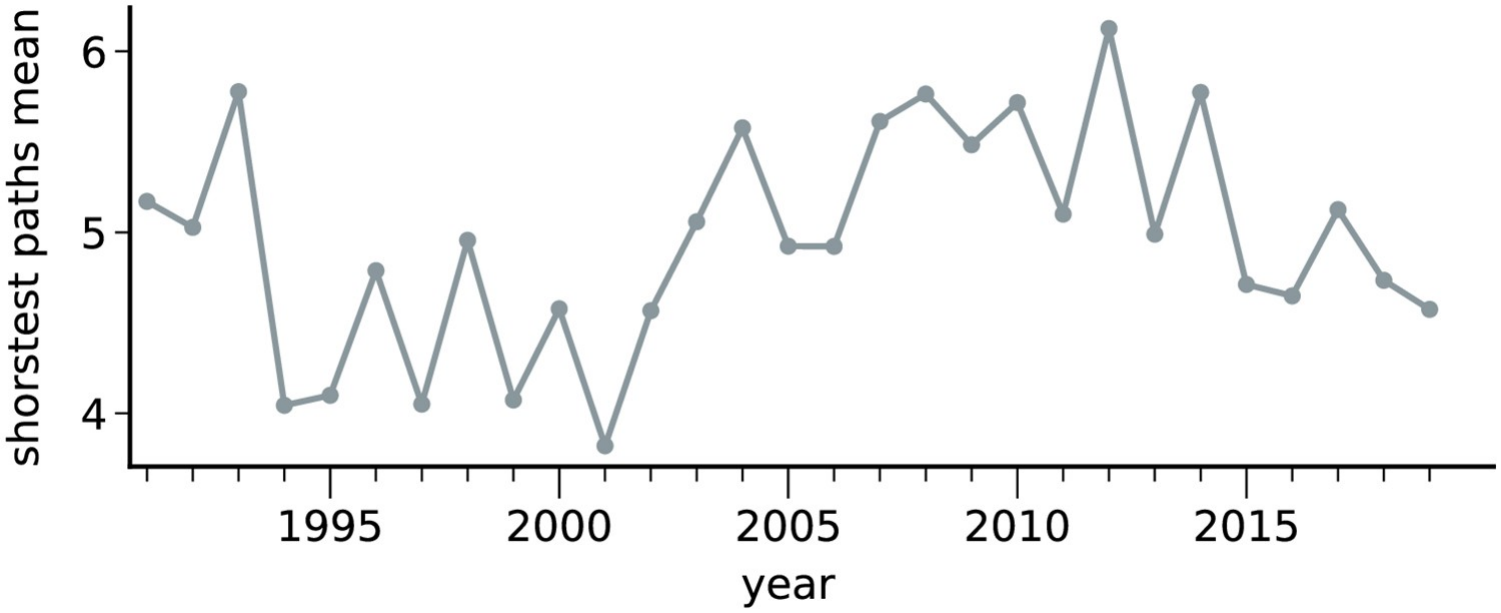
Evolution of the average shortest path length. While the average distance ranges in the interval 4 ≤ *d*_*G*_ ≤ 6, the networks obtained in the interval between 1994 and 2002 are typically more cohesive than the ones obtained between 2003 and 2012.

The global evolution of the obtained political networks is depicted in Fig 5, where colors represent political parties. An interactive visualization of these networks is available online at https://filipinascimento.github.io/politiciannetworks. The first important property that can be recovered from the networks is the global *cohesiveness*. This can be computed using a measurement that quantifies the distance between two nodes (deputies) in the network. If deputies vote in a similar fashion, one expects that the average distance between them is short. The obtained evolution of the global distances (*d*_*G*_ in Eq 9) is shown in Fig 6. All distances typically range in the interval 4 ≤ *d*_*G*_ ≤ 6. Two patterns, however, can be identified. In the period between 1994 and 2002, distances are in the interval 4 ≤ *d*_*G*_ ≤ 5. Conversely, in the period between 2003 and 2014, the average distances typically are typically higher, in the interval 5 ≤ *d*_*G*_ ≤ 6. Interestingly these two intervals correspond to opposite parties occupying the presidency. This observation might indicate that different government ideologies may affect the way in which deputies vote.

**Fig 7:**
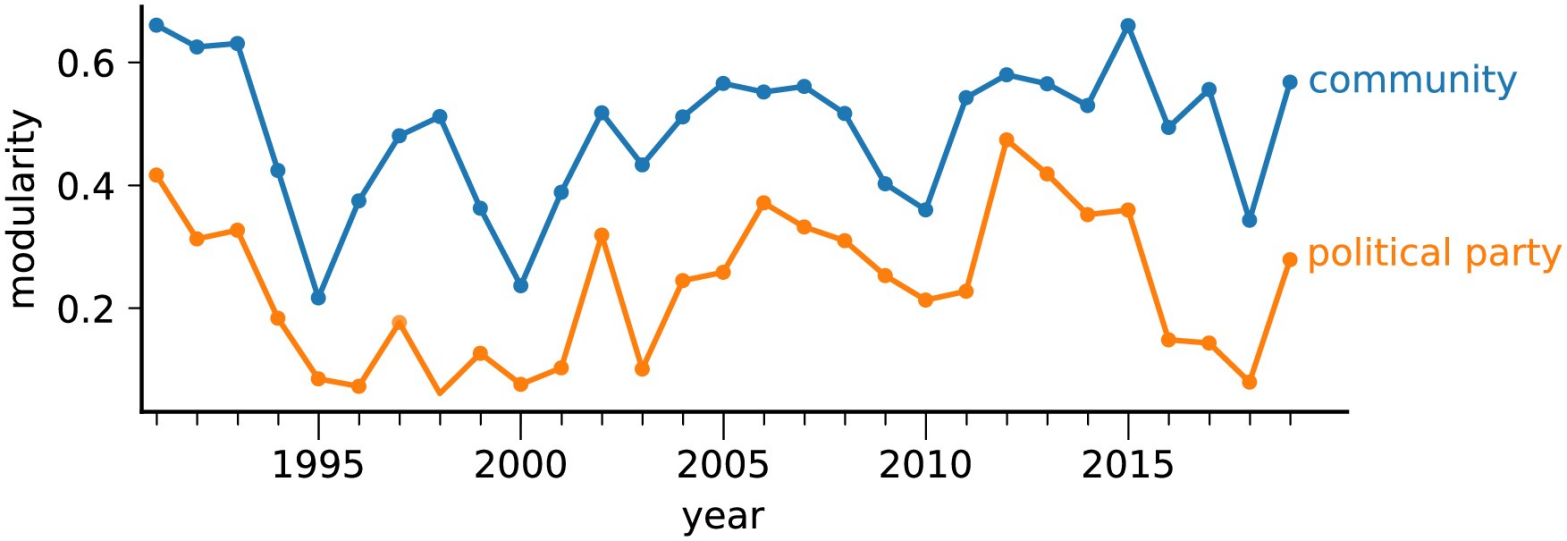
Evolution of the modularity measurement. To compute the modularity, we considered the groups obtained via community detection method (blue curve) and the organization of deputies in political parties (orange curve).

The *consistency* of political parties can be measured by probing whether groups defined via political parties are consistent with network communities, which are obtained using only the connectivity of the network. Network communities are obtained via modularity optimization and, for this reason, this method tends to cluster nodes into the same community if they are strongly connected inside the community, with a few external links. The evolution of the quality of the clustering obtained via network communities and political parties are shown in Fig 7. The modularity of the obtained network communities outperforms the modularity of political parties. This means that there is a high tendency of deputies of different political parties to be connected. While the difference in modularity seems to be roughly constant along time, it is possible to observe higher deviations in particular periods. This is apparent e.g. in the years 2016 and 2017, which coincides with a period of post-impeachment political transition in Brazil. In both years, only PT was found to form an independent community, according to Fig 5. An opposite effect occurred in 2015, when the difference in modularity reached its minimum value. This is evident in Fig 5, because political parties are consistent with network communities.

**Fig 8:**
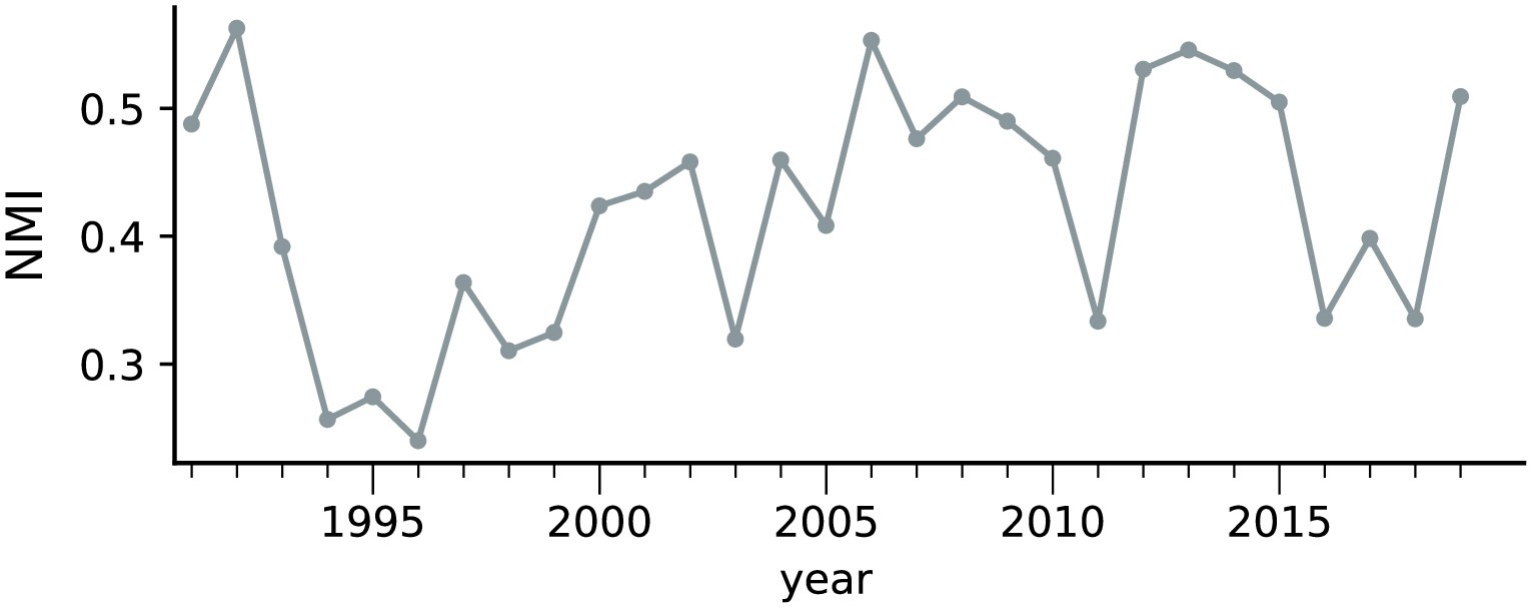
Normalized Mutual Information (NMI) between communities and political parties. A major transition affecting the NMI occurred just before the first PSDB presidency, from 1993 to 1994.

The divergence between network communities and political parties can be further analyzed via Normalized Mutual Information (NMI) [37], which measures the quality of the obtained partitions (network communities) based on a set of nodes membership labels (political parties). The evolution of the NMI index is shown in Fig 8. Overall, the correspondence between political parties and communities varies along time, but a major rupture seems to occurs just before the first PSDB term (1994) and just before impeachment (2016). Notably, the highest discrepancy (lowest value of NMI) occurred in 1996, which is in accordance with the visualization provided in Fig 5. This result confirms that, in the Brazilian political scenario, in most years the natural organization of deputies in communities has a low correspondence with party organization.

**Fig 9:**
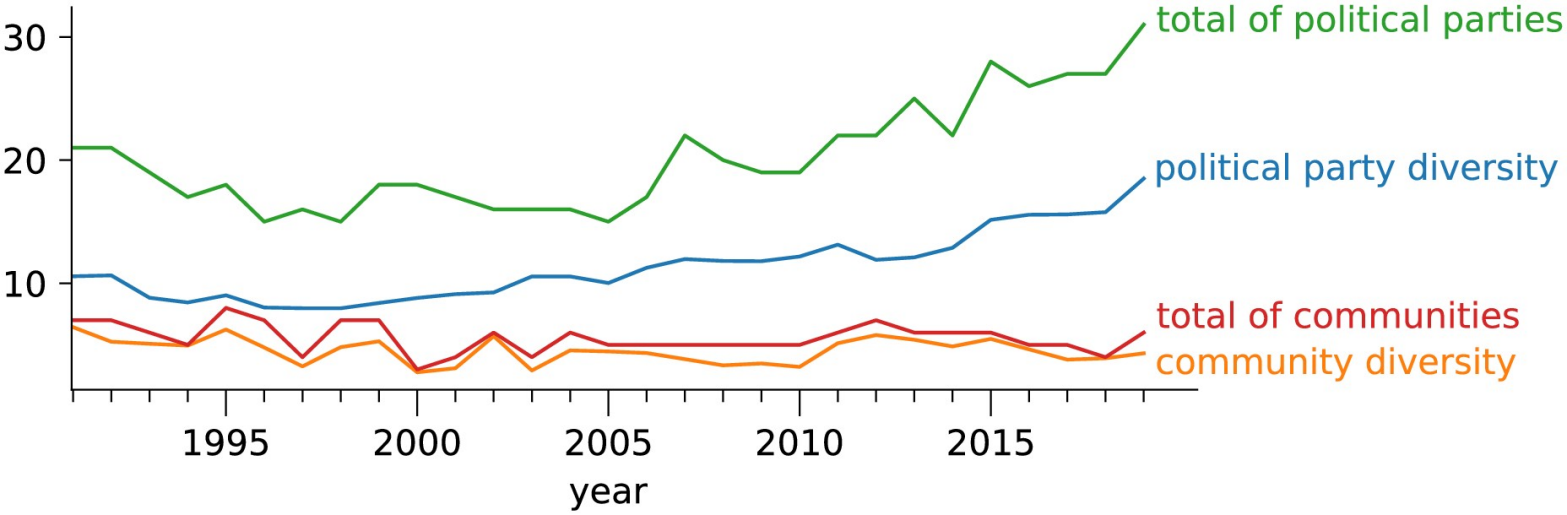
Size evolution of political parties and network communities. Even though the Brazilian Chamber of Deputies is represented by 29 political parties, the effective number of network communities (community diversity) in 2019 is only 3.37.

The discrepancy between political parties and network communities is also evident when one studies the variability in parties/communities size using the definition of effective number of groups (see Eq 7). The *effective* number of parties and communities was computed using the true diversity of the distribution of parties/communities size, as defined in Eq 7. The results are depicted in Fig 9. Since 2005, an increase in the total number of parties (green curve) is clear. In 2019, the Brazilian Chamber of Deputies is made up of roughly 29 political parties. The effective number of parties (blue curve), however, reveals the existence of only 20 political parties. Surprisingly, the topology of the network revealed the existence of only about 5 communities of deputies. In other words, even though 29 political parties exist, *D*(communities) ≃ 3.37 (see Eq 7) in 2019. In other words, only 3.37 communities are effectively present in the Brazilian political scenario in 2019.

The small effective number of parties computed via diversity is not a trivial result. Even though there are only 3 possible results in a single voting, the number of votings is large enough to create several clusters (network communities) of similar voting behavior, as displayed in Fig 5. The effective number of groups considers both the number and size of the obtained network communities. Because only a few communities comprise most of the deputies, the effective number of groups is much smaller than the total number of political parties.

### Mesoscopic analysis

The obtained networks can also shed light into the understanding of the political dynamic at the mesoscopic level. An interesting analysis concerns the quantification of the pairwise distance between political parties along time. This analysis is important, e.g. to quantify the level of *coalision* (or *opposition*) between parties and party *isolation*. Such concepts are oftentimes used in politics because abrupt or recurrent changes in these quantities may precede a relevant change in the political scenario.

**Fig 10:**
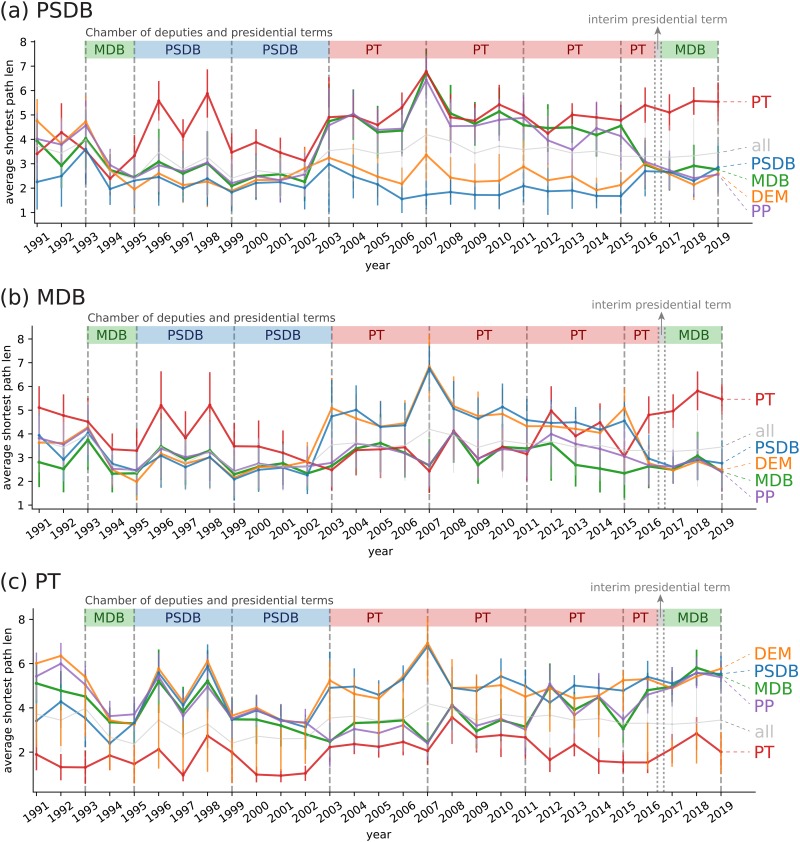
Average shortest path length between (a) PSDB, (b) MDB and (c) PT deputies to other parties. Distances are computed using Eq 8. The fragmentation (e.g. *d*(PSDB, PSDB)) is computed using Eq 12. Presidential terms are highlight in the period between 1993 and the end of 2018.

In Fig 10(a), we show the temporal evolution of the distances between PSDB and other parties. The distances are computed using Eq 8. In this figure, we also show presidential terms in different colors, including the transition post-impeachment led by MDB presidency (2016–2018, green background). Interestingly, along the years of PSDB presidency, PT was found to be the most distant party from PSDB. This is consistent with the traditional sentiment of polarization between PT and PSDB in these years. During PT presidency, we note that PT and PSDB were still distant, however, two additional parties (MDB and PP) were found to increase (and keep) a larger distance from PSDB. This is consistent with the real political scenario, since MDB and PT shared a *coalition* along the period characterized by PT presidency. These observations are consistent with the visualization provided in Fig 5.

Another interesting pattern that can be extracted from Fig 10(a) concerns the evolution of PSDB fragmentation (i.e. *F*(PSDB) = *d*(PSDB, PSDB)), as defined in Eq 12. The PSDB fragmentation corresponds to the blue curve. Note that the typical distance between PSDB deputies is compatible with the distance between PSDB and DEM virtually along all considered years and, markedly, before 2002. Such a proximity between these two parties is also evident from the visualization of the network structure. A strong concordance with MDB and PP is also apparent before 2002 and after 2016. We can also observe that the distance curves among parties seem to correlate for certain periods of time. For instance, between 1993 and 2002, the curves for PSDB, MDB, DEM and PP are very similar; while from 2003 to 2011, the group consisting of PT, MDB and PP vary together. This is another indication of strong coalition between these parties during that time period.

In Fig 10(b), we show the distances between MDB and other parties. In this case, the transition in government seems to play a prominent role in the behavior of this party. Between 1991 and 2001, the only party distant from MDB is PT. Conversely, between 2002 and 2011, PT and MDB became much closer. This is a consequence of the the political coalition shared by these parties along these years. Differently from the traditional alliance in previous years, MDB dissociated itself from PSDB and DEM between 2002 and 2016 (see also Fig 5). The rapprochement between these parties took place only in 2016, in the post-impeachment transition. The MDB fragmentation along time is roughly constant, however, apart from 2012-2015, the fragmentation is always similar to the typical distance between MDB and the closest parties. We also found that shortest path curves for PP (not shown) are similar to those obtained for MDB.

The distance between PT and other political parties is depicted in Fig 10(c). It is clear from the data the PT and PSDB are continuously distant from each other since 1995. During PSDB presidency, the isolation of PT is clear: no other party is closer to PT than PT itself. In the first years of PT presidency, PT became closer to other parties, including MDB and PP. Some years before a new transition in governments, however, PT became distant from all others, specially after 2015. This isolation scenario became consistent even after the presidential impeachment (2016).

**Fig 11:**
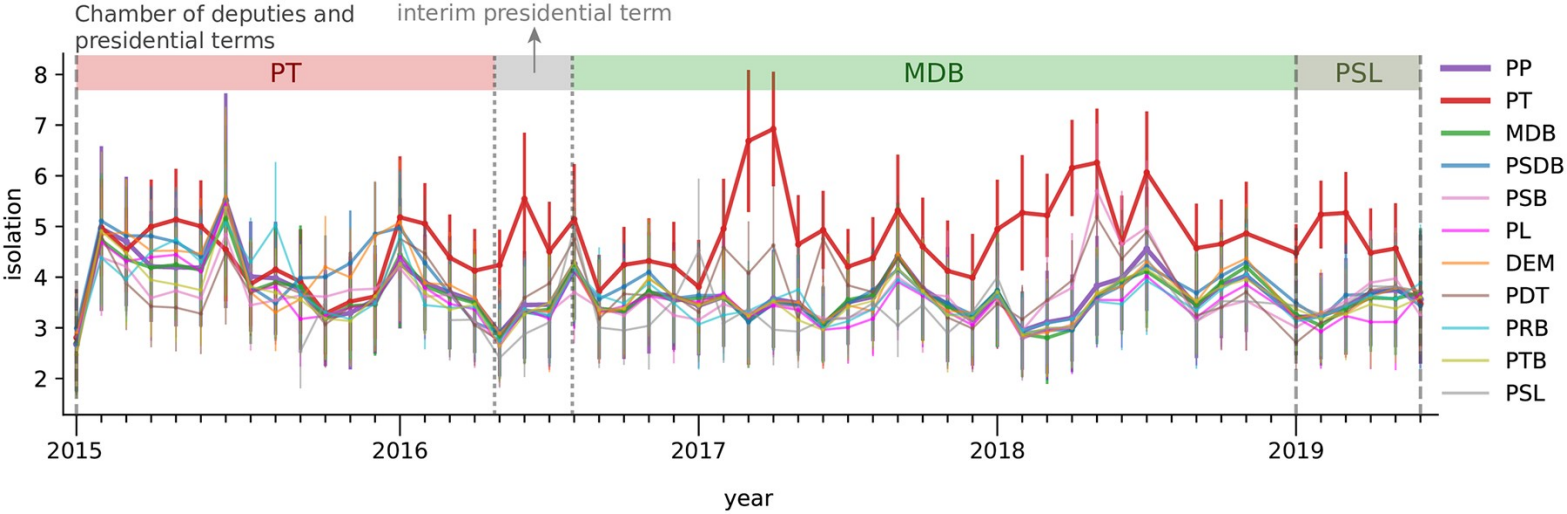
Isolation of political parties few months before and after impeachment. The isolation of parties is computed using Eq 11. PT turned out to be the most isolated party a few months before impeachment. The relative isolation became stronger after 2017.

In Fig 11, we analyze the *isolation* of political parties few months before and after the impeachment of the Brazilian President, Dilma Rousseff (PT). PT and MDB terms are represented in red and green background colors, respectively. The transition between these two presidencies is shown in gray background color. Apart from PT, all parties have similar values and patterns of isolation along time. While PT isolation was compatible with many the behavior displayed by many other parties, its isolation increased (relatively to other parties) months before impeachment. During the first months of the interim presidential term, PT isolation increased relatively to other parties. After 2017, PT turned out to be the most isolated political party. PT isolation is also clear in the visualization provided in Fig 5, specially in 2017. A sudden change in relative isolation months before impeachment could indicate the vulnerability of the party, and potentially a signal of a major change in the political scenario.

### Parties distribution in communities

Even though the Brazilian political system comprises several parties, only 3 to 5 effective communities where detected from the vote correlation networks. In this section, we study how parties are distributed among these communities. This is illustrated by focusing on two political events: the elections and part of the first term in office of Luiz Inácio Lula da Silva (PT) from 2000 to 2003; and the years preceding and after the impeachment of Dilma Rousseff (PT).

**Fig 12:**
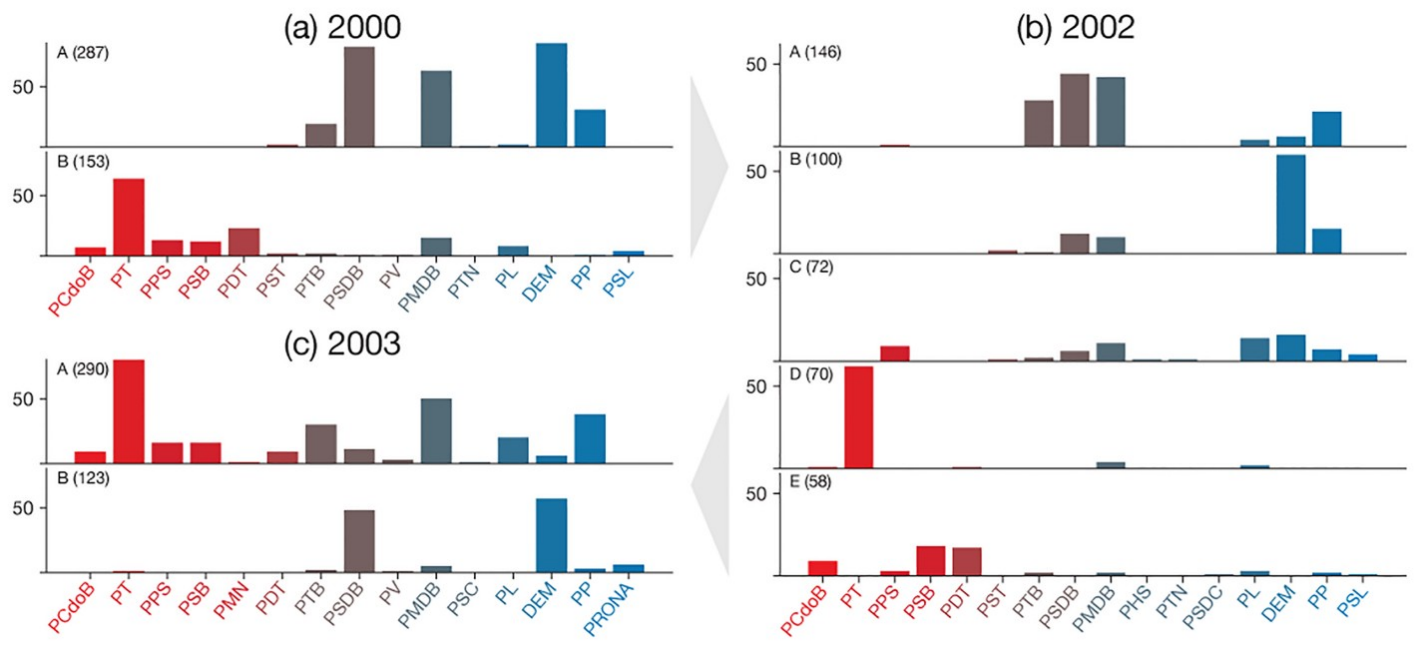
Distribution of parties among communities for Mr. Silva’s first presidential term and preceding elections. The charts correspond to the communities obtained for the indicated year. Each bar chart indicate a community with the number of deputies indicated in parenthesis. Only communities with at least 15 deputies are considered. Parties were ordered from left to right (and colored from red to blue) according to their political positions [39]. The names of parties and abbreviations are historical and were not updated to the currently adopted names.

Fig 12 shows the parties distribution among the communities for networks corresponding to three years. In this figure, parties are ordered from left to right (and colored from red to blue, respectively) according to their political positions. During the elections, in 2000 (a), only two major communities are present, with *A* corresponding to the right-wing and central parties and *B* to the left-wing parties. The system, however, changes to a more complicated distribution of parties in 2002 (b), having central and right-wing parties belonging to different communities. A single community, *D*, encompass all deputies from the presidential party (PT) at that time. Interestingly, in 2003 (c), the coalition of the government party is consolidated, giving rise to a community (*A*) comprising parties from the left, central and right wings. Political oppositions constitute a single community of two parties, PSDB and DEM.

**Fig 13:**
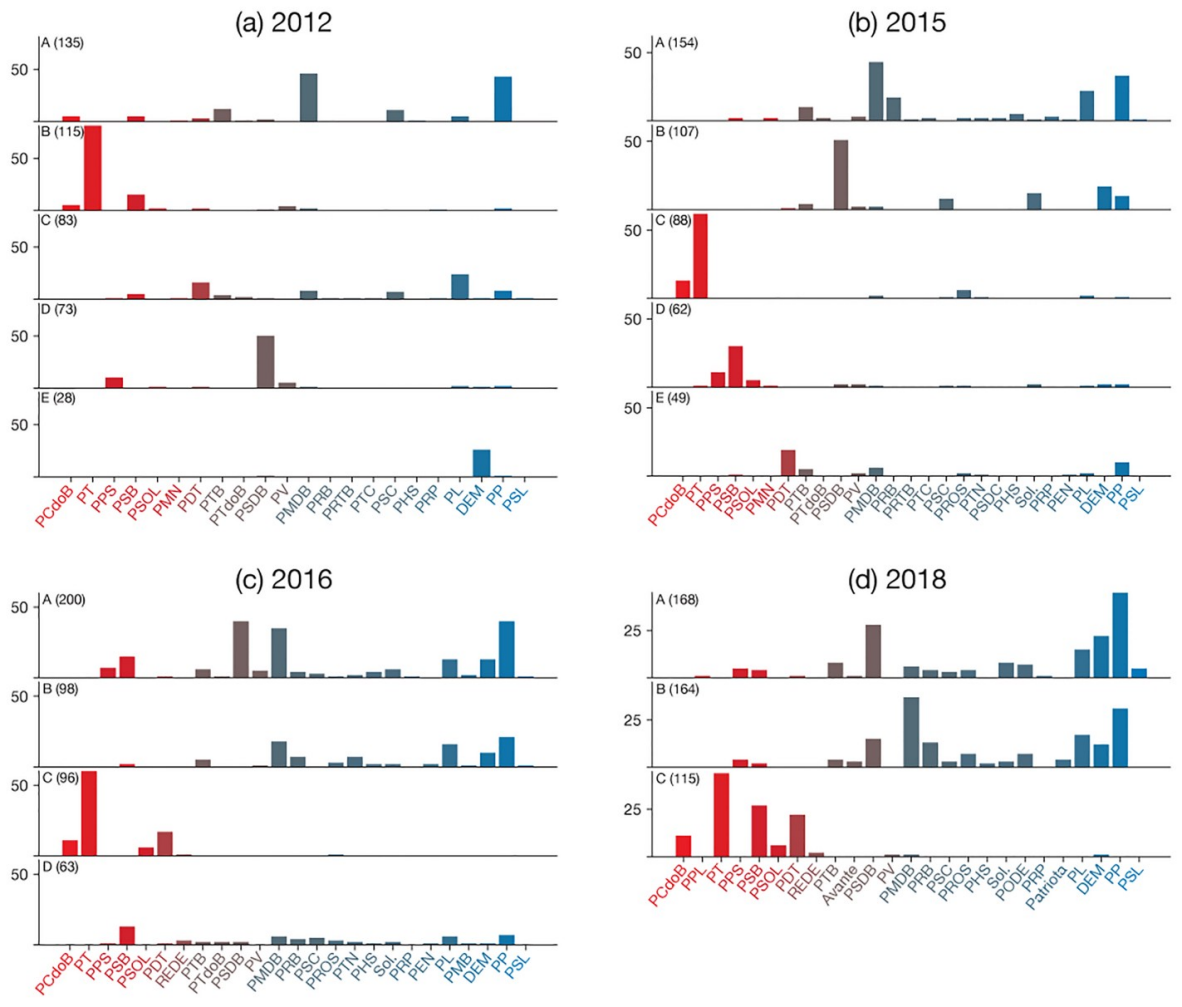
Distribution of parties among communities before and after the impeachment of Dilma Rousseff. The charts are organized in a similar fashion as those shown in Fig 12.

The distributions of parties found in 2003 remained similar through all the Mr. da Silva’s presidency period until 2011, corresponding to the beginning of his successor’s, Dilma Roussef, first term. Fig 13 shows the parties distributions for two periods predating the impeachment process, 2012 (a) and 2015 (b). In 2012 (a), the collision formed during Mr. da Silva’s first and second terms does not incorporate as many parties as before, also leading to more communities. Three years later (b), corresponding to the period of the first part of the impeachment process; the central and right-wing parties are spread along two communities *A* and *B*, with the left-wing also divided in at least two major communities with PT almost isolated in community *C*.

In 2016 (Fig 13), during the impeachment process and Michel Temer’s (PMDB) presidency, a new government coalition is established comprising most of the center and right-wing parties. PT and other left-wing parties embody community *C*, corresponding to the main political opposition block. More recently, in 2018 (d), the distribution did not change significantly, except that the left-wing parties now belong to the same communities.

## Conclusion

In a democratic nation, the political organization can reflect its cultural context, economic and social interactions. The understanding of the role of deputies and parties alike is essential to improve any democratic system. Here we proposed a framework to assist the understanding of the dynamics of political parties. The relationship between deputies in the lower chamber was represented as a network, where two deputies are linked if they voted in a similar way in different propositions. Using concepts borrowed from network science, we defined politics concepts such as *isolation*, *fragmentation* and *coalition*, which were measured in terms of topological distances.

Several interesting results were found when the proposed framework was applied to analyze the Brazilian Chamber of Deputies. During PSDB term, PT was found to be the most distant party from PSDB and vice-versa. The coalition shared by parties was easily identified from both distance metrics and the visualization of the obtained networks. The networks displayed a modular topology, however, we found a low degree of consistency when comparing topological groups and political parties in most years. Surprisingly, we found that even though the Brazilian political scenario is highly fragmented, only a few topological groups emerge. While in 2019 the lower chamber comprised 29 distinct parties, we found that *effectively* there are roughly only 3 communities of deputies, according to the diversity measurement. A detailed analysis in the period between 2015 and 2019 revealed that PT isolation increased a few months before the presidential impeachment of Dilma Rousseff (PT). This could be an early sign before the impeachment proceedings against former president Rousseff effectively started.

We also found that the communities obtained for the vote correlation networks can have a diverse range of patterns and most of times do not seem to be defined solely based on party’s ideology. For instance, during Luiz Inácio Lula da Silva’s presidency, the community structure captured the government coalition and the opposition, with the first encompassing parties of all political positions.

As future work, we intend to use additional information to analyze and possibly predict the results of votes based on the history of deputies, parties. This could be accomplished by using textual data obtained from the propositions and transcripts from deputies’ discourses. Another interesting path of analysis would be studying the party coalition dynamics under a theoretical framework. For instance by building a model that takes into into consideration the time correlations or causal relationships between the empirical time series.

While the focus of this manuscript was in identifying and quantifying relevant political quantities via network science, we advocate that this framework could also be used in other scenarios. An analogous approach could be used to analyze other types of social networks with similar characteristics, i.e. entities with pre-defined labels that interact in a complex manner. This is the case of the *Science of Science* field [38], where authors interact via collaborations and, at the same time, they can be labeled according to diverse criteria: country, university, research field/group or others.
